# Adiponectin and the Control of Metabolic Dysfunction: Is Exercise the Magic Bullet?

**DOI:** 10.3389/fphys.2021.651732

**Published:** 2021-04-07

**Authors:** Lauretta I. Otu, Akaninyene Otu

**Affiliations:** ^1^Department of Health, Faculty of Health Sciences, Liverpool Hope University Liverpool, Liverpool, United Kingdom; ^2^Department of Internal Medicine, College of Medical Sciences, University of Calabar, Calabar, Nigeria

**Keywords:** adiponectin, physical activity, metabolism, exercise, inflammation

## Physical Activity and Metabolic Health—the Wider Benefits

Throughout history, movement was absolutely essential—humans moved to survive—but this has evolved with the comforts provided by modern society limiting movement to sport and leisure rather than for survival (Exercise metabolism, 2017). This evolution has promoted sedentary behavior and given rise to the global pandemic of physical inactivity with deleterious effects on humankind. Physical inactivity is estimated to be the fourth leading risk factor for global mortality (6% of deaths globally) (World Health Organization, 2009). There is compelling evidence that physical activity promotes cardiorespiratory fitness and lowers risk for developing several chronic medical illnesses such as cardiovascular disease, obesity, diabetes mellitus, and specific cancers, in particular breast and colon cancer (Macera and Powell, 2001; Macera et al., 2003; Warburton et al., 2006). In May 2004, the fifty-seventh World Health Assembly endorsed Resolution WHA57.17: Global Strategy on Diet, Physical Activity and Health that urged Member States to develop national physical activity action plans and policies to increase the physical activity levels of their citizens (World Health Organization, 2004). Since then, giant strides have been made at national and global levels to tackle physical inactivity by adopting novel strategies such as developing guidelines and providing physical environments which support safe active commuting recreational activity.

Long term health benefits have been linked to physical activity and the inverse relationship between physical activity and mortality has been established. Obesity has reached epidemic proportions globally and has nearly tripled worldwide since 1975. According to the World Health Organization (WHO) estimates, there were more than 1.9 billion adults, aged 18 years and older, who were overweight in 2016 of which 650 million were obese (World Health Organization, 2020). Lower levels of obesity (Slentz et al., 2009; Hopps and Caimi, 2011) insulin resistance, blood lipid levels (Kraus et al., 2002), hypertension (Kelly and Kelly, 2001), and improved cardiovascular fitness (Duscha et al., 2005) have been linked with regular exercise.

## Adiponectin—An Important Metabolic Biomarker

Physical activity impacts on the body's biological systems through its effect on numerous biomarkers. One such biomarker is the hormone adiponectin which is exclusively secreted in adipose tissues (Hu et al., 1996). Discovered in 1995, adiponectin is abundant in the circulation with plasma levels ranging from 3 to 30 μg/ml (Arita et al., 1999; Ouchi et al., 2003). Adiponectin is key in metabolic disorders like insulin resistance (Lim et al., 2008), as well as inflammation (Ouchi and Walsh, 2007). Inflammation is closely linked with several diseases that threaten human health (Dandona et al., 2004). There is evidence that a causal relationship exists between inflammation and diseases like obesity (Engstrom et al., 2003; Wellen and Hotamisligil, 2005), insulin resistance (Shoelson et al., 2006), type two diabetes mellitus (Wellen and Hotamisligil, 2005), and cardiovascular diseases (Engstrom et al., 2004).

Inflammation has equally been linked to some cancers with increased risk of malignancies closely related to chronic inflammation (Ekbom et al., 1990; Gulumian, 1999). A wide range of anti-inflammatory activities have been attributed to the hormone adiponectin (Ouchi and Walsh, 2007). These range from a reduction in the expression of adhesion molecules (Ouchi et al., 1999; Kobashi et al., 2005), to the inhibition of pro-inflammatory cytokine production (Ajuwon and Spurlock, 2005). Adiponectin is also involved in the induction of anti-inflammatory factors (Tsatsanis et al., 2005).

Adiponectin occurs as three basic isoforms in circulation: the high molecular weight (HMW), moderate molecular weight (MMW), and low molecular weight (LMW) adiponectin (Neumeier et al., 2006; Stein et al., 2007). These isoforms activate isoform specific pathways whilst still exerting a common effect on monocyte cells (Neumeier et al., 2006). Monocyte cells are central to development of obesity and cardiovascular diseases owing to their ability to secrete pro-inflammatory cytokines (Neumeier et al., 2006). Structurally, adiponectin has a carboxyl terminal comprising the globular domain, a mutable section and an amino tail which makes up the collagen-like domain. By binding to specific receptors, adiponectin is able to exert its effect in the human body. Present in most tissues is the adiponectin receptor 1 (AdipoR1) which has a high propensity to bind to the full length adiponectin whereas the adiponectin 2 (AdipoR2), mostly found in the liver, has an equal but moderate affinity for both the full length and globular adiponectin molecules (Yamauchi et al., 2003).

The relationship between exercise and circulating levels of adiponectin has been of interest to researchers over the years. The debate on the value of exercise on adiponectin is ongoing as some studies have recorded improvements in adiponectin levels following exercise (Kraemer et al., 2003; Hsieh and Wang, 2005; Bluher et al., 2006; Jürimäe et al., 2006; Oberbach et al., 2006) while a number of studies have failed to record any improvements in values following exercise (Hulver et al., 2002; Ryan et al., 2003; Fergusson et al., 2004; Nassis et al., 2005; Punyadeera et al., 2005; Jamurtas et al., 2006). Several key functions have been clearly associated with the hormone adiponectin. The results of a meta-analysis that showed that low serum adiponectin level increased the risk of a first cardiovascular event in the Han Chinese population (Zhang et al., 2012) does highlight the need for further evaluation of the role of adiponectin in health and disease.

## Adiponectin and Insulin Resistance—The Molecular Basis

The association of adiponectin with insulin resistance was first established by Yamauchi and colleagues in 2001 when reduced adiponectin levels and insulin resistance was observed in mouse models with altered insulin sensitivity (Yamauchi et al., 2001). By inhibiting the expression of hepatic gluconeogenic enzymes and the rate of endogenous glucose production, adiponectin has been found to sensitize the body to insulin (Combs et al., 2001). The insulin sensitizing effect of adiponectin is also thought to be brought about by an increase in fatty acid oxidation. This is most likely mediated through the activation of adenosine monophosphate-activated protein kinase (AMPK) by the globular adiponectin (Yamauchi et al., 2003).

AMPK activation is believed to cause the phosphorylation of Acetyl-CoA Carboxylase beta (ACC-β) which then inhibits the activity of the enzyme Acetyl CoA Carboxylase (ACC) leading to a decline in Malonyl CoA content. As a result, the activity of carnitine palmitoyl transferase is suppressed and fatty acid oxidation is increased (Hardie et al., 1998; Arita et al., 1999; Winder and Hardie, 1999). Activation of AMPK may stimulate β-oxidation and glucose uptake (Yamauchi et al., 2002), resulting in the binding adiponectin to its AdipoR1 receptor. With the binding of the hormone to the AdipoR2 receptor, there is the activation of PPAR-α signaling pathways, which increases fatty acid oxidation and energy utilization. Consequently, the triglyceride content of the liver and skeletal muscle is decreased resulting in improved insulin sensitivity. This is a desirable effect as insulin resistance predisposes people to type 2 diabetes (Manson et al., 1991).

Continuous build-up of intra hepatic triglyceride content has been shown to be closely linked with a deterioration of insulin action in the liver, skeletal muscle and adipose tissue (Korenblat et al., 2008). Likewise, intramuscular triglyceride (IMTG) content has also been linked with insulin resistance (Goodpaster et al., 1997) with increased levels of IMTG resulting in insulin resistance (Pan et al., 1997; Krssak et al., 1999).

The increase in fatty acid oxidation, glucose metabolism and the raised insulin sensitivity that results from the activation of AMPK by adiponectin has also been linked to the hormone's angiogenic property (Maeda et al., 2002). The resultant surge in phosphatidylinositol 3-kinase—Akt signaling (Yamauchi et al., 2001) within the muscle is likely responsible for the stimulation of angiogenic growth factor synthesis (Takahashi et al., 2002). Angiogenic growth factors are thought to play a crucial role in several physiological processes like wound healing. In myocardial infarction, they stimulate the growth of collaterals to ischaemic tissue (Risau, 1990; Goncalves, 2000).

Exercise has been shown to increase insulin sensitivity (Corcoran et al., 2007; Hawley and Lessard, 2007; Parker et al., 2016). One of the putative mechanisms is through increases in plasma adiponectin levels (Kriketos et al., 2004; Lim et al., 2008). In a recent study conducted on male rats, the improvement in insulin resistance was shown to be mediated via the binding of the hormone to the adiponectin receptor 1 (AdipoR1) (Cho et al., 2015).

## Adiponectin and Inflammation

The anti-inflammatory property of adiponectin has been linked to its ability to increase the secretion of anti-inflammatory protein interleukin 10 (IL10) and interlukin1 receptor (IL 1) (Wolf et al., 2004). When in abundance, these anti-inflammatory agents result in suppressed release of inflammatory cytokines from activated monocyte cells (Wolf et al., 2004). The hormones' binding to its AdipoR1 receptor mediates this effect (Yamaguchi et al., 2005).

Studies have shown that inflammatory biomarkers are lower in people who are involved in frequent and more intense physical activity (King et al., 2003). The drop in inflammatory markers is thought to occur simultaneously with increase in anti-inflammatory substances like IL-10 whose secretion is increased in the presence of adiponectin (De Lemos et al., 2012).

## Adiponectin and the Vasculature

An intact endothelium is vital for health. Central to a healthy endothelium is nitric oxide (NO) which acts through various mechanisms to prevent the degeneration of the endothelium (Moncada and Higgs, 2006). Adiponectin has been shown to enhance the gene expression of endothelium nitric oxide synthase (eNOS): the enzyme responsible for the synthesis of NO (Chen et al., 2003; Hattori et al., 2003). Adiponectin activates AMP kinase which increases the activity of eNOS resulting in increases in NO production (Goldstein and Scalia, 2004).

## Exercise and Circulating Adiponectin Levels

Experimental and clinical data on the effects of acute exercise on adiponectin level is not robust. It has been shown that some of the benefits of exercise accrue from acute relatively brief sessions of exercise (Haskell and Wolffe, 1994). To examine if this holds true for adiponectin, Kraemer et al. in 2003 looked at the impact of 30 min of heavy continuous running on adiponectin levels in healthy male as well as the impact of intermittent exercise in well-trained runners. In both instances, adiponectin values remained unchanged. A single session of submaximal exercise failed to yield any positive changes in adiponectin concentration when overweight males ran for 45 min at 65% of VO_2max_ (Jamurtas et al., 2006). Likewise, plasma adiponectin concentrations did not change in either males or females who cycled for 60 min at 65% of VO_2max_ (Fergusson et al., 2004).

In contrast to findings of the above mentioned studies, adiponectin levels were seen to be elevated after abdominally fat men exercised at either low or high intensity, with plasma concentrations rising in both instances (Saunders et al., 2012). Similarly, adiponectin was altered positively after maximal acute exercise in highly trained athletes (Jürimae et al., 2005) and after a 30-min rowing exercise by male athletes at their individual anaerobic threshold (75.2 ± 2.9% of VO_2max_). These confounding results could be explained by individual variability in adiponectin concentration (Jürimäe et al., 2006).

More recently, a meta-analysis of 14 randomized controlled trials conducted among 347 youth revealed that exercise was associated with a significant increase in adiponectin; exercise intensity, change in body fat, total exercise programme duration, as well as duration of the sessions were all found to significantly influence the effect of exercise on adiponectin (García-Hermoso et al., 2017). The authors concluded that exercise seems to increase adiponectin levels in childhood obesity. Another systematic review and meta-analysis of 22 trials with 2,996 individuals showed that physical exercise and, specifically, aerobic exercise resulted in higher adiponectin and lower leptin levels in prediabetic and diabetic adults (Becic et al., 2018). Interestingly, a study using 2-month-old Wistar rats showed a reduction adiponectin protein levels in serum but there were no significant differences in Adiponectin receptor 1 (AdipoR1) gene expression in either muscle group studied following intense or moderate exercise (Jiménez-Maldonado et al., 2019). Whilst there is a good number of studies looking at the impact of aerobic exercise on adiponectin values, research relating to the effect of resistance exercise on adiponectin are few. The impact of resistance exercise on adiponectin levels has been examined by two studies. One of these studies recorded increases in adiponectin levels after a single bout of resistance exercise in both trained weight lifters and in people who combined weight lifting and running (Varady et al., 2010). In the study by Varady and colleagues, adiponectin values went up by 30 ± 7% and ± 9% in response to acute weight training. In contrast to this, the study by Mansouri and colleagues did not record any changes in adiponectin levels following a single bout of exercise (Mansouri et al., 2011).

## Effect of Diet and Race on Adiponectin Levels

There is evidence linking diet and race to plasma adiponectin levels. In a study involving mice, a decrease of adiponectin expression was selectively observed in white adipose tissue (WAT) of mice fed a normal-vitamin A, high-fat diet and those fed a high-vitamin A diet (Landrier et al., 2017). A recent review of 16 articles revealed that the consumption of saturated fat reduced the levels of adiponectin in animal models, while in humans, the consumption of healthy and Mediterranean diets were positively associated with adiponectin levels (Reis et al., 2010). Cipryan and colleagues recently showed that in healthy young individuals consuming a very low-carbohydrate, high-fat while performing regular exercise over a 12-week period produced a significant (and beneficial) increase in adiponectin and a significant decrease in leptin levels (Cipryan et al., 2021).

Adiponectin is encoded by the ADIPOQ gene located on chromosome 3q27. In a study involving both black and white women that examined polymorphisms in ADIPOQ, ADIPOR1, and ADIPOR2 in relation to adiponectin levels and body mass index (BMI), SNP rs17366568 in ADIPOQ was significantly associated with serum adiponectin levels in white women only (Cohen et al., 2011). Further evidence for the influence of race on adiponectin levels is provided by a population-based study involving 29,000 participants that showed that adiponectin levels were lower among blacks and Hispanics (Gardener et al., 2013). Further investigation is required to more clearly describe the effect of diet and race on adiponectin levels.

## Conclusion

Physical activity in the form of exercise is an important integrative therapy in metabolic, immunologic and chronic diseases. Exercise has been shown to affect the levels of the hormone adiponectin. Adiponectin mediates many biological effects and has a role in several cellular processes such as proliferation, inflammation, and oxidative stress. More research is need to elucidate the interconnections between adiponectin and various forms of physical exercise to optimize the potential metabolic benefits of performing exercise.

## Author Contributions

LO and AO conceived the manuscript. LO wrote the first draft. AO reviewed the manuscript. All authors read and approved the final manuscript.

## Conflict of Interest

The authors declare that the research was conducted in the absence of any commercial or financial relationships that could be construed as a potential conflict of interest.
